# The heart and HIV therapy: exploring the dual cardiovascular impact of antiretroviral treatment – a narrative review

**DOI:** 10.1097/MS9.0000000000003465

**Published:** 2025-06-10

**Authors:** Chukwuka Elendu, Daniel E. Otobrise, George S. Blewusi, Nzubechukwu A. Iheanetu, Precious A. Olaofe, Nwamaka C. Bob-Ume, Jamila A. Suleiman, Jemilah I. Hassan, Barakat I. Ibikunle, Peace Mordi, Deborah M. Olabode, Kehinde M. Ogungbade, Funmilayo G. Adelakun, Kris N. Idion, Sa’Adatu I. Kure, Adaobi S. Ikedilo, Grace D. Ogunkoya, Abolore Aminat Ajakaye, Adaeze B. Eze, Obinna D. Nwaizuzu

**Affiliations:** a Federal University Teaching Hospital, Owerri, Nigeria; b University of Medical Sciences, Ondo, Nigeria; c Johns Hopkins Bloomberg School of Public Health, Baltimore, Maryland, USA; d V. N. Karazin Kharkiv National University, Kharkiv, Ukraine; e Kyiv Medical University, Kyiv, Ukraine; f All Saints University School of Medicine, St Vincent & The Grenadines; g Ivano-Frankivsk National Medical University, Ivano-Frankivsk, Ukraine; h Nahda University, Beni Suef, Egypt; i University of Szeged, Albert Szent-Györgyi Medical School, Szeged, Hungary; j Gannan Medical University, Ganzhou, Jiangxi, China; k Babcock University, Ilishan-Remo, Nigeria; l Afe Babalola University, Ado-Ekiti, Nigeria; m Delta State University Teaching Hospital, Oghara, Nigeria; n American University of Antigua, St. John's, Antigua; o Piedmont Athens Regional Medical Centre, Athens, Georgia, USA; p Bogomolets National Medical University, Kyiv, Ukraine; q Regions Healthcare Hospitals and Specialist Clinics, Mgbrichi, Nigeria; r University of Debrecen, Debrecen, Hungary; s University of Nigeria, Nsukka, Nigeria

**Keywords:** ART, cardiovascular disease, endothelial dysfunction, HIV, inflammation, myocardial involvement

## Abstract

Human immunodeficiency virus (HIV) is a primary global health concern, affecting an estimated 38 million individuals as of 2023. While HIV is best known for its immunosuppressive effects, its cardiovascular implications are equally significant. HIV infection contributes to cardiovascular disease (CVD) through chronic immune activation, systemic inflammation, endothelial dysfunction, oxidative stress, and coagulopathy. These mechanisms increase the risk of myocardial infarction, cardiomyopathy, heart failure, pulmonary hypertension, and thrombotic events in people living with HIV (PLWH). However, the advent of Highly Active Antiretroviral Therapy (HAART) has transformed HIV from a fatal illness into a chronic, manageable condition. Despite this progress, HAART – particularly regimens involving protease inhibitors and certain nucleoside reverse transcriptase inhibitors – has introduced new cardiovascular challenges. These include dyslipidemia, insulin resistance, mitochondrial toxicity, and altered adipose tissue distribution, all of which further elevate cardiovascular risk.

Additionally, HAART modifies immune and metabolic pathways, potentially exacerbating underlying HIV-associated cardiovascular mechanisms. Emerging research into microbial translocation and gut dysbiosis also points to complex interactions between HIV, HAART, and cardiovascular outcomes. As survival improves for PLWH, cardiologists and infectious disease specialists must collaborate to address both the direct effects of HIV and the unintended cardiovascular consequences of its treatment. The authors’ review explores the dual impact of HIV and HAART on the cardiovascular system, emphasizing the importance of integrated care, early risk assessment, and therapeutic strategies tailored to the unique cardiovascular profile of PLWH.

**HIGHLIGHTS**

- HIV drugs help control the virus but may harm the heart.
- Some treatments raise cholesterol and affect insulin levels.
- Monitoring heart health in HIV care is essential.

## Introduction and background

HIV, the Human Immunodeficiency Virus, is a major global health challenge with profound implications for nearly every organ system, including the cardiovascular system. First identified in the early 1980s, HIV has since become a pandemic, with approximately 38 million people living with the infection worldwide as of 2023^[1-3]^. While advances in treatment, particularly the advent of Highly Active Antiretroviral Therapy (HAART), have transformed HIV from a fatal disease into a manageable chronic condition, emerging evidence highlights the long-term complications associated with both the virus and its treatment^[4-6]^.

Among these complications, cardiovascular disease (CVD) has garnered increasing attention due to its impact on morbidity and mortality among individuals living with HIV. HIV infection itself is intrinsically linked to an increased risk of cardiovascular complications. The virus initiates a chronic state of immune activation and systemic inflammation, which persists even with effective viral suppression via HAART^[7]^. Elevated levels of inflammatory markers such as interleukin-6 (IL-6) and C-reactive protein (CRP) have been consistently observed in HIV-positive individuals and are strongly associated with endothelial dysfunction, accelerated atherosclerosis, and myocardial injury^[8]^. Furthermore, the direct effects of the virus on cardiomyocytes and vascular endothelial cells contribute to a range of cardiac pathologies, including myocarditis, dilated cardiomyopathy, and pericardial effusion^[5]^.

The authors’ review does not claim that HIV-related cardiovascular complications are entirely novel or absent in medical reference books. Instead, it aims to synthesize recent evidence – much of which has emerged after the publication of major textbooks – on the evolving relationship between HIV, HAART, and cardiovascular health. The goal is to provide an updated, thorough, and clinically relevant summary of the pathophysiological mechanisms, clinical manifestations, and management challenges posed by cardiovascular complications in people living with HIV. Additionally, the review highlights areas where new research findings may not yet be reflected in standard medical texts, particularly concerning the cardiovascular impact of newer HAART regimens, the role of immune activation, and the influence of coinfections.

Given the increasing life expectancy of HIV-positive individuals, cardiovascular disease (CVD) has become a leading cause of morbidity and mortality in this population. Our paper is therefore both timely and necessary, as it integrates current scientific literature to support improved understanding and care of cardiovascular disease in the context of HIV.

### Data collection

We adopted a narrative review approach to synthesize current literature on the cardiovascular impact of HIV and HAART. Relevant studies were identified through searches of PubMed, Google Scholar, Embase, and the Cochrane Library for articles published between January 2000 and December 2023, using combinations of keywords and MeSH terms such as “HIV,” “HAART,” “cardiovascular complications,” and related terms. Only peer-reviewed studies focusing on adult populations and addressing the pathophysiology, clinical presentation, or management of cardiovascular conditions in the context of HIV and/or HAART were included. The findings were narratively summarized to highlight key mechanisms, clinical insights, and areas for further research.

### Prevalence of cardiovascular disease in HIV

The global burden of cardiovascular disease (CVD) among people living with HIV (PLWH) has significantly increased over the past few decades. Advances in antiretroviral therapy (ART), particularly Highly Active Antiretroviral Therapy (HAART), have transformed HIV from a fatal disease into a manageable chronic condition. However, as life expectancy increases in PLWH, cardiovascular complications have emerged as a leading cause of morbidity and mortality, especially in regions with access to HAART. Global estimates suggest that the prevalence of CVD among PLWH ranges from 6% to 15%, varying widely based on geography, age, and socioeconomic factors^[1,2]^. In high-income countries, where the availability of HAART is nearly universal, the prevalence of CVD among PLWH is strongly influenced by metabolic complications associated with long-term HAART use, such as dyslipidemia and insulin resistance. Studies in the United States and Europe report that PLWH are approximately 50% more likely to develop ischemic heart disease compared to their HIV-negative counterparts^[3]^. In these settings, traditional cardiovascular risk factors, including smoking, obesity, and sedentary lifestyles, often compound HIV-specific risks, leading to a greater incidence of coronary artery disease and myocardial infarction^[4]^.

In contrast, low- and middle-income countries (LMICs), where over 70% of people living with HIV (PLWH) reside, present a unique epidemiological pattern. In sub-Saharan Africa, for instance, the predominant cardiovascular complications in PLWH include cardiomyopathies and pericardial diseases rather than ischemic heart diseases, reflecting a greater burden of untreated opportunistic infections and limited access to advanced healthcare services^[5,6]^. Rheumatic heart disease remains a significant contributor to cardiovascular morbidity in this population, underscoring the role of poverty and inadequate healthcare infrastructure^[7]^. Additionally, delayed initiation of highly active antiretroviral therapy (HAART) and intermittent drug supply in LMICs often exacerbate HIV-associated inflammation and immune activation, further increasing cardiovascular risk^[8]^. Demographic factors also play a critical role in shaping cardiovascular outcomes in PLWH. Age is a significant determinant, with older adults experiencing a disproportionately higher burden of cardiovascular disease (CVD). This is partly due to the cumulative effects of HAART over time and the natural aging process, which accelerates in PLWH due to persistent low-grade inflammation^[9,10]^. Gender disparities are also evident, with some studies suggesting that HIV-positive women may have a higher relative risk of cardiovascular events compared to HIV-negative women than men do in similar comparisons^[11]^. Hormonal differences and sociocultural factors, such as limited access to healthcare and economic empowerment, may contribute to these disparities^[12]^. Socioeconomic status is another critical determinant of cardiovascular health in PLWH. Individuals from lower socioeconomic backgrounds often face barriers to accessing consistent and comprehensive HIV care, including regular cardiovascular screening and management of traditional risk factors. In LMICs, financial constraints often limit the availability of second and third-line HAART regimens, forcing patients to rely on older drugs associated with greater cardiotoxicity^[13,14]^. Conversely, in high-income settings, despite better access to healthcare, disparities persist among racial and ethnic minorities, who often face structural inequities that increase their vulnerability to both HIV and cardiovascular diseases^[15,16]^.

### Pathophysiology of HIV and Cardiovascular Impact

**Figure 1.**
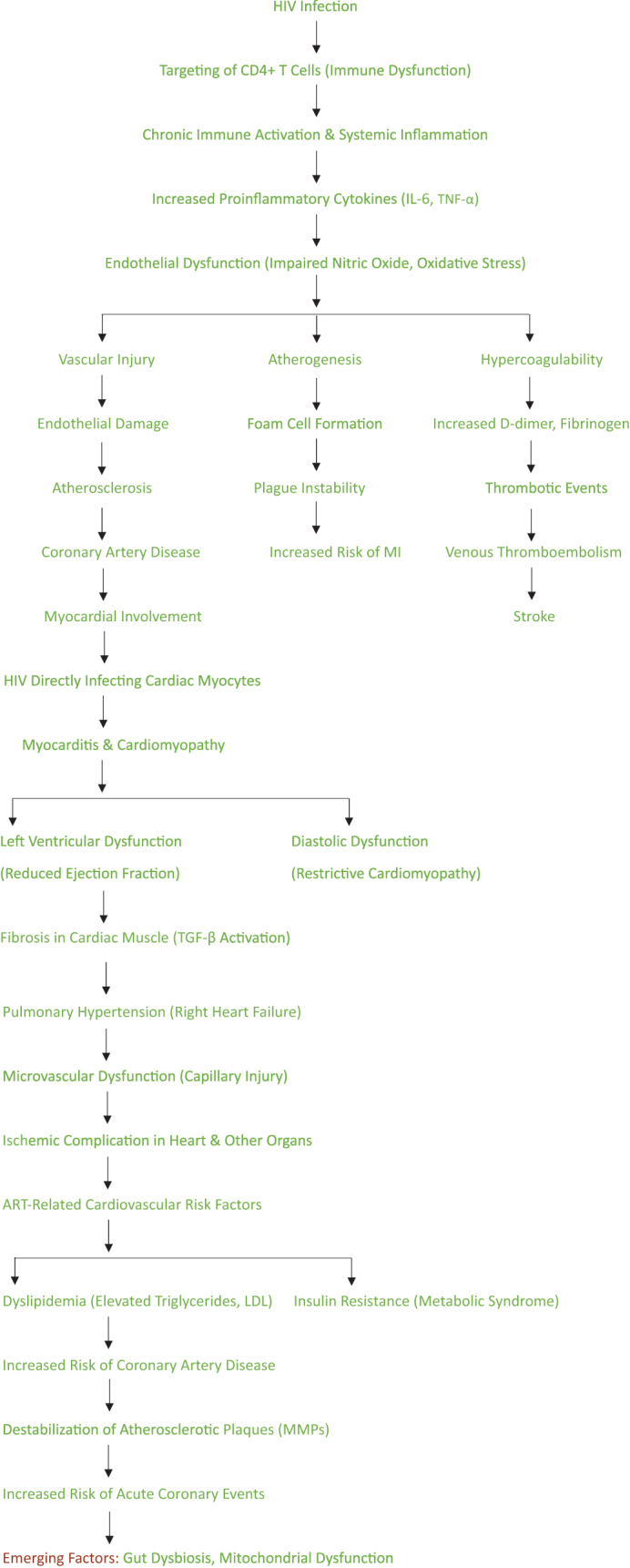
Pathophysiology of HIV-related cardiovascular disease.

HIV primarily targets CD4 + T cells, leading to immune dysfunction and progressive immunosuppression. The depletion of these cells disrupts immune homeostasis, resulting in chronic immune activation and systemic inflammation, even in patients with suppressed viral loads on ART. This persistent inflammation is a significant driver of cardiovascular diseases (CVD) in PLWH^[2]^. Proinflammatory cytokines, such as interleukin-6 (IL-6) and tumor necrosis factor-alpha (TNF-α), remain elevated in HIV-infected individuals, contributing to endothelial dysfunction, vascular injury, and atherogenesis^[17-19]^. Endothelial dysfunction is a hallmark of HIV-related cardiovascular pathology. HIV proteins, such as Tat and Nef, directly interact with endothelial cells, disrupting their normal functions. These proteins promote oxidative stress, reduce nitric oxide bioavailability, and impair endothelial repair mechanisms. Such alterations predispose individuals to atherosclerosis, hypertension, and other vascular diseases^[4]^. Moreover, the chronic activation of monocytes and macrophages in PLWH enhances the production of reactive oxygen species (ROS) and promotes foam cell formation, accelerating the development of atherosclerotic plaques^[20-22]^. HIV infection is also associated with hypercoagulability, further increasing the risk of thrombotic events. Elevated levels of D-dimer, fibrinogen, and tissue factor have been observed in HIV-infected individuals, reflecting an ongoing prothrombotic state. This hypercoagulability is linked to endothelial injury and platelet activation, both exacerbated by chronic inflammation^[6]^. Consequently, PLWH are at an increased risk of venous thromboembolism, myocardial infarction, and stroke compared to the general population^[23-25]^. Myocardial involvement is another critical aspect of HIV-related cardiovascular disease (see Fig. 1, which illustrates the pathophysiological progression from HIV infection to cardiovascular complications, including myocarditis, coronary artery disease, and cardiomyopathy, and highlights the impact of antiretroviral therapy). HIV can directly infect cardiac myocytes, leading to myocarditis and subsequent cardiomyopathy. This condition is often characterized by left ventricular dysfunction, reduced ejection fraction, and heart failure. Opportunistic infections, such as toxoplasmosis and cytomegalovirus, further contribute to myocardial damage^[26-28]^. Additionally, the chronic inflammatory state in HIV promotes fibrosis within the cardiac muscle, leading to diastolic dysfunction and restrictive cardiomyopathy. Studies have shown that elevated levels of transforming growth factor-beta (TGF-β) in PLWH contribute to myocardial fibrosis, underscoring the role of inflammation in cardiac remodeling^[9]^. Pulmonary hypertension is another cardiovascular complication frequently observed in PLWH. The pathogenesis of HIV-associated pulmonary hypertension involves the interplay of direct viral effects, endothelial dysfunction, and inflammation. HIV proteins induce pulmonary artery smooth muscle cell proliferation and promote vasoconstriction, increasing pulmonary vascular resistance. Over time, this results in right ventricular hypertrophy and eventual right heart failure^[10]^. Despite the benefits of ART in suppressing viral replication and improving survival, it has been implicated in the development of cardiovascular complications. Protease inhibitors (PIs), a key component of ART, have been associated with dyslipidemia, insulin resistance, and increased visceral adiposity, all of which are risk factors for atherosclerosis. Similarly, nucleoside reverse transcriptase inhibitors (NRTIs), such as stavudine and zidovudine, have been linked to mitochondrial toxicity and endothelial dysfunction^[11]^. ART-induced dyslipidemia is characterized by elevated levels of triglycerides, low-density lipoprotein (LDL) cholesterol, and reduced high-density lipoprotein (HDL) cholesterol. This lipid profile significantly increases the risk of coronary artery disease (CAD) in PLWH. Furthermore, insulin resistance induced by PIs contributes to metabolic syndrome, further exacerbating cardiovascular risk^[12]^. HIV-related immune activation also plays a role in the destabilization of atherosclerotic plaques, increasing the likelihood of acute coronary events. Activated macrophages and T cells infiltrate the plaques, releasing matrix metalloproteinases (MMPs) that degrade the fibrous cap. This process makes the plaques more prone to rupture, resulting in thrombosis and myocardial infarction^[13]^. In addition to its effects on large vessels, HIV impacts the microvasculature, contributing to microvascular dysfunction. Reduced capillary density and impaired autoregulation have been observed in HIV-infected individuals, leading to ischemic complications in various organs, including the heart. This microvascular dysfunction is believed to result from a combination of endothelial cell injury, perivascular inflammation, and oxidative stress^[14]^. Emerging evidence suggests that HIV-related gut dysbiosis may also contribute to cardiovascular complications. Disrupting gut mucosal integrity allows microbial translocation into the systemic circulation, amplifying immune activation and inflammation. Markers of microbial translocation, such as lipopolysaccharide (LPS) and soluble CD14 (sCD14), are associated with increased cardiovascular risk in PLWH^[15]^. Another critical factor in the pathophysiology of HIV-related cardiovascular disease is mitochondrial dysfunction. HIV infection and certain ART regimens impair mitochondrial function, leading to increased oxidative stress and apoptosis. Mitochondrial damage within endothelial cells and cardiomyocytes further exacerbates vascular and myocardial injury, highlighting the importance of mitochondrial health in cardiovascular outcomes^[16]^.

This flowchart illustrates the key steps in the pathophysiology of HIV-related cardiovascular disease, starting from the infection to the development of various cardiovascular complications such as myocarditis, coronary artery disease, and cardiomyopathy, as well as the impact of ART – source: Authors’ Creation.

### Cardiovascular complications associated with HIV

One of the most notable cardiovascular complications associated with HIV is myocarditis, an inflammatory condition affecting the heart muscle. Myocarditis in people living with HIV (PLWH) is often due to direct viral invasion or secondary to opportunistic infections, such as cytomegalovirus (CMV) or toxoplasmosis, which are commonly seen in immunocompromised individuals. HIV itself can infect cardiac myocytes, leading to direct cytopathic effects and eliciting an immune response that exacerbates myocardial damage^[1,29,30]^. Patients with HIV-related myocarditis often present with symptoms such as chest pain, arrhythmias, and heart failure. Diagnosing myocarditis can be challenging, as the symptoms overlap with other cardiac conditions. Endomyocardial biopsy, though invasive, remains the gold standard for diagnosis, but noninvasive imaging techniques like cardiac MRI are increasingly being utilized for their diagnostic accuracy^[12]^. The management of HIV-related myocarditis involves treating the underlying infection, optimizing antiretroviral therapy (ART), and managing heart failure symptoms with standard therapies.

Pericarditis, characterized by inflammation of the pericardium, is another significant cardiovascular complication in PLWH. In the pre-ART era, pericarditis was a common manifestation of advanced HIV infection, frequently associated with opportunistic infections such as tuberculosis (TB), fungal infections, and bacterial sepsis^[13]^. Even in the ART era, TB remains a leading cause of pericarditis in HIV-endemic regions, particularly in sub-Saharan Africa. HIV-related pericarditis often presents with symptoms such as sharp chest pain, dyspnea, and pericardial effusion, which can progress to cardiac tamponade if untreated. Echocardiography is the cornerstone of diagnosis, allowing visualization of pericardial effusion and assessment of hemodynamic compromise. Treatment strategies focus on addressing the underlying etiology, such as initiating anti-TB therapy in cases caused by Mycobacterium tuberculosis, alongside supportive measures like corticosteroids for severe inflammatory responses^[14]^.

Cardiomyopathy, particularly dilated and hypertrophic forms, is a well-documented complication in people living with HIV (PLWH). Dilated cardiomyopathy (DCM) is more common and is often linked to chronic inflammation, immune activation, and direct viral effects on cardiac myocytes. HIV-associated DCM is also associated with micronutrient deficiencies, such as selenium, which impair myocardial function^[15]^. Symptoms include fatigue, dyspnea, and reduced exercise tolerance, while echocardiography typically reveals global ventricular dilation and reduced ejection fraction. Hypertrophic cardiomyopathy, though less common, has been reported in HIV patients and may result from antiretroviral therapy (ART)-related toxicities or genetic predisposition. ART plays a dual role in cardiomyopathy: while it reduces the systemic inflammatory burden of HIV, certain antiretroviral drugs, particularly protease inhibitors, have been implicated in myocardial toxicity. Managing HIV-related cardiomyopathy involves a multidisciplinary approach, combining ART optimization with standard heart failure therapies, including angiotensin-converting enzyme (ACE) inhibitors, beta-blockers, and diuretics^[16,29,30]^.

**Figure 2.**
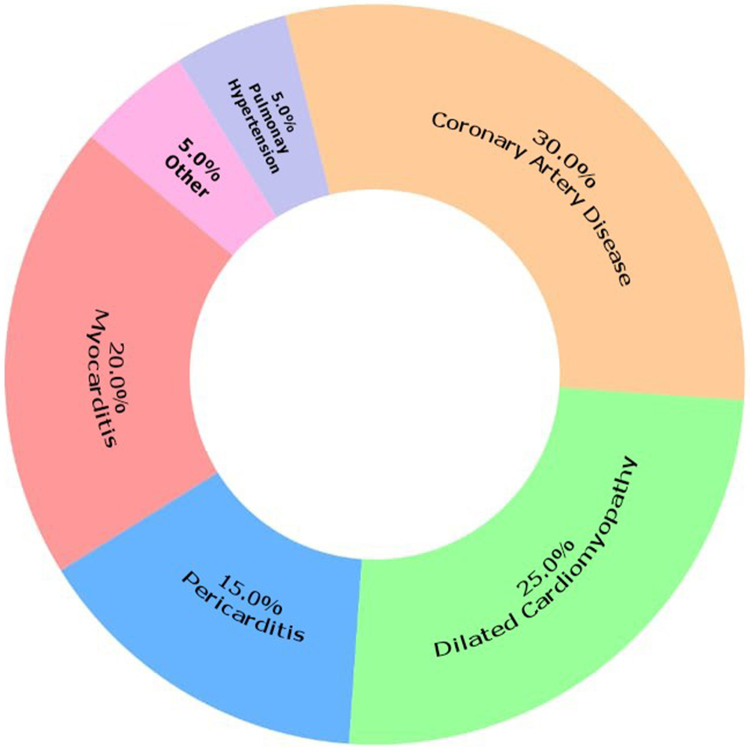
Prevalence of cardiovascular complications in HIV-infected individuals.

Coronary artery disease (CAD) is emerging as a leading cause of morbidity and mortality in people living with HIV (PLWH), reflecting the interplay of traditional cardiovascular risk factors, HIV-related immune activation, and antiretroviral therapy (ART)-associated metabolic changes (see Fig. 2, which illustrates the prevalence of various cardiovascular complications in HIV-infected individuals, including myocarditis, pericarditis, dilated cardiomyopathy, coronary artery disease, pulmonary hypertension, and other complications, with each segment labeled by percentage for easy visualization). HIV infection is associated with chronic inflammation and immune activation, accelerating atherosclerosis through endothelial dysfunction, oxidative stress, and increased plaque formation^[7]^. Moreover, ART, particularly older regimens with protease inhibitors, contributes to metabolic derangements such as dyslipidemia, insulin resistance, and central obesity, further increasing CAD risk^[8]^. Studies have shown that PLWH have a higher prevalence of subclinical atherosclerosis and coronary calcifications compared to the general population, even after adjusting for traditional risk factors. Clinically, CAD in PLWH may present as acute coronary syndromes or chronic stable angina. Diagnostic approaches include coronary angiography, CT angiography, and stress testing, while management follows standard guidelines with medical therapy, revascularization, and aggressive risk factor modification. Emerging strategies, such as statin therapy for primary prevention, are gaining traction in this population^[9]^.

This pie chart illustrates the prevalence of various cardiovascular complications in HIV-infected individuals. The data include myocarditis, pericarditis, dilated cardiomyopathy, coronary artery disease, pulmonary hypertension, and other complications. Each segment is labeled with its percentage for easy visualization. Source: Authors’ creation.

Pulmonary hypertension (PH) is another significant cardiovascular complication of HIV, with a reported prevalence of 0.5% in people living with HIV (PLWH), which is significantly higher than in the general population. HIV-associated PH is classified as Group 1 pulmonary arterial hypertension (PAH) and is thought to result from a combination of endothelial dysfunction, chronic inflammation, and direct viral effects on the pulmonary vasculature^[10]^. Patients often present with nonspecific symptoms such as dyspnea, fatigue, and syncope, leading to delays in diagnosis. Echocardiography is the initial diagnostic tool for assessing pulmonary artery pressures, but right heart catheterization remains the gold standard for confirming the diagnosis and guiding management. Treatment options include antiretroviral therapy (ART) to control HIV replication alongside specific therapies for PAH, such as endothelin receptor antagonists, phosphodiesterase-5 inhibitors, and prostacyclin analogs^[11]^.

Gender and age-related differences in cardiovascular complications among PLWH are increasingly recognized. Women living with HIV face distinct cardiovascular risks, including a heightened susceptibility to myocardial infarction and stroke, which may be exacerbated by hormonal fluctuations during menopause and underrepresentation in clinical studies^[29]^. Estrogen deficiency, coupled with chronic immune activation and the metabolic effects of ART, may accelerate vascular aging in this subgroup. Additionally, as the HIV population ages, there is a notable rise in age-associated cardiovascular disorders such as atherosclerosis, heart failure, and arrhythmias. Aging with HIV is complicated by cumulative ART exposure, persistent inflammation, and polypharmacy, all of which contribute to adverse cardiac outcomes^[30]^. These factors necessitate gender- and age-specific screening strategies and tailored therapeutic approaches.

Traditional cardiovascular risk factors, including smoking, obesity, hypertension, and diabetes, play a critical role in exacerbating the cardiovascular burden in people living with HIV (PLWH). Smoking rates are disproportionately high in this population, with studies reporting prevalence rates of up to 40% among HIV-positive individuals compared to 15% in the general population^[12]^. Smoking exacerbates endothelial dysfunction and inflammation, compounding the cardiovascular risk associated with HIV. Obesity and metabolic syndrome, which are becoming increasingly prevalent in PLWH due to prolonged antiretroviral therapy (ART) use and aging, further contribute to cardiovascular complications. ART-associated lipodystrophy, characterized by central fat accumulation and peripheral fat loss, is a key driver of metabolic syndrome in this population^[13]^. Hypertension and diabetes, common comorbidities in PLWH, also amplify the risk of cardiovascular events. Importantly, the interplay between traditional risk factors and HIV-specific mechanisms creates a synergistic effect, accelerating cardiovascular disease progression.

### Role of HAART in Cardiovascular Health

The introduction of HAART has effectively suppressed viral replication, reducing immune activation and systemic inflammation. These effects are critical in mitigating the direct and indirect cardiovascular damage caused by HIV. In untreated HIV infection, chronic inflammation plays a pivotal role in endothelial dysfunction, atherogenesis, and myocardial damage. By suppressing viral replication, HAART reduces markers of inflammation, such as C-reactive protein (CRP) and interleukin-6 (IL-6), thereby ameliorating the inflammatory burden on the cardiovascular system^[3,4]^.

Despite these benefits, HAART has been associated with several adverse cardiovascular effects, which can complicate the overall health outcomes of PLWH. Certain classes of antiretroviral drugs, particularly protease inhibitors (PIs) and some nucleoside reverse transcriptase inhibitors (NRTIs), have been implicated in the development of dyslipidemia, insulin resistance, and other metabolic derangements (see Table 1, which summarizes HAART drug classes, their mechanisms of action, standard dosages, adverse effects, cardiovascular implications, and notable interactions). Dyslipidemia, characterized by elevated triglycerides, low-density lipoprotein cholesterol (LDL-C), and reduced high-density lipoprotein cholesterol (HDL-C), is a common side effect of HAART. These lipid abnormalities contribute to the accelerated development of atherosclerosis, increasing the risk of myocardial infarction and stroke in PLWH^[5,6]^.

**Table 1:** HAART Drug Classes and Their Mechanisms

Drug Class	Examples	Mechanism of Action	Target Protein/Enzyme	Administration Route	Standard Dosage	Adverse Effects	Cardiovascular Implications	Notable Interactions
**Nucleoside Reverse Transcriptase Inhibitors (NRTIs)**	Zidovudine (AZT), Lamivudine (3TC), Abacavir (ABC), Tenofovir (TDF), Emtricitabine (FTC)	Inhibits reverse transcriptase by causing premature DNA chain termination.	Reversetranscriptase	Oral	AZT: 300 mg BID; TDF: 300 mg/day	Lactic acidosis, hepatotoxicity, lipodystrophy	Abacavir: Increased risk of myocardial infarction.	Avoid nephrotoxic drugs (e.g., NSAIDs for TDF).
**Non-Nucleoside Reverse Transcriptase Inhibitors (NNRTIs)**	Efavirenz (EFV), Nevirapine (NVP), Etravirine (ETR), Rilpivirine (RPV).	Bind non-competitively to reverse transcriptase and inhibit its function.	Reverse transcriptase	Oral	EFV: 600 mg/day; NVP: 200 mg BID	Rash, hepatotoxicity,neuropsychiatric symptoms	Efavirenz: Dyslipidemia, potentially pro-atherogenic	Rifampin reduces efficacy; avoid taking it with high-fat meals (RPV).
**Protease Inhibitors (PIs)**	Atazanavir (ATV), Darunavir (DRV), Lopinavir/Ritonavir (LPV/r)	Inhibits protease, preventing the cleavage of viral polyproteins.	HIV protease	Oral	DRV: 800 mg/day + RTV: 100 mg/day	Dyslipidemia, insulin resistance, GI disturbances	Dyslipidemia and increased cardiovascular risk	Interact with CYP3A4 substrates and inhibitors.
**Integrase Strand Transfer Inhibitors (INSTIs)**	Dolutegravir (DTG), Raltegravir (RAL), Bictegravir (BIC), and Elvitegravir (EVG)	Block integrase to prevent viral DNA integration into host DNA.	HIV integrase	Oral	DTG: 50 mg/day; RAL: 400 mg BID.	Insomnia, weight gain, headache	Low cardiovascular risk, emerging metabolic risks.	Antacids reduce absorption; avoid divalent cations.
**Entry Inhibitors**	Maraviroc (MVC), Enfuvirtide (T-20)	Prevent HIV entry into CD4 + cells by blocking CCR5 or fusion.	CCR5 (MVC)/GP41 (T-20)	MVC: Oral; T-20: SC	MVC: 300 mg BID; T-20: 90 mg BID	Injection site reactions (T-20), hepatotoxicity	Minimal cardiovascular impact	CYP3A inhibitors increase MVC levels
**Post-Attachment Inhibitors**	Ibalizumab (IBA)	Monoclonal antibody blocking HIV-1 from binding CD4 receptors.	CD4 receptor	IV infusion	800 mg every 2 weeks	Infusion-related reactions, rash	Limited data; low cardiovascular risk	None significant
**Pharmacokinetic Enhancers**	Ritonavir (RTV), Cobicistat (COBI)	Inhibit CYP3A to boost plasma levels of other antiretroviral drugs.	CYP3A	Oral	RTV: 100 mg/day; COBI: 150 mg/day	GI upset, dyslipidemia, drug interactions	May exacerbate dyslipidemia and metabolic issues.	Potent CYP3A inhibitors: major drug interaction risk
**Fusion Inhibitors**	Enfuvirtide (T-20)	Prevent HIV from fusing with host cell membranes.	GP41	Subcutaneous	90 mg BID	Injection site reactions, bacterial pneumonia	Minimal cardiovascular impact	None significant
**CCR5 Antagonists**	Maraviroc (MVC)	Blocks CCR5 co-receptor, preventing HIV entry.	CCR5	Oral	300 mg BID	Hepatotoxicity, dizziness	Rare cases of myocardial ischemia	Interacts with CYP3A substrates

Table 1 includes nine columns covering critical pharmacological information. The Drug Class column categorizes the major HAART classes, such as NRTIs, NNRTIs, PIs, INSTIs, and fusion inhibitors. Examples provide representative drugs, like zidovudine for NRTIs and efavirenz for NNRTIs. The Mechanism of Action explains how each class inhibits HIV replication by blocking reverse transcriptase or integrase. The Target Protein/Enzyme specifies viral components, such as reverse transcriptase or protease. The Administration Route highlights how drugs are delivered, mainly orally but also via injections for some. The Standard Dosage includes typical regimens, such as 300 mg twice daily for zidovudine. Adverse Effects cover side effects, such as lactic acidosis for NRTIs or lipid abnormalities for PIs. Cardiovascular Implications explore impacts like dyslipidemia and hypertension, often linked to PIs. Notable Interactions list interactions, such as PIs inhibiting CYP3A4, which affects co-administered drugs. This structure clarifies HAART’s clinical and pharmacological aspects – source: Authors’ Creation.

Insulin resistance and the subsequent development of diabetes mellitus are other significant concerns associated with HAART. Drugs such as zidovudine (AZT) and stavudine (d4T) have been linked to mitochondrial toxicity, which impairs glucose metabolism. Moreover, PIs such as lopinavir/ritonavir exacerbate this risk by altering fat distribution and promoting visceral adiposity. These metabolic changes not only increase the risk of diabetes but also contribute to systemic inflammation, further elevating cardiovascular risk^[7,8]^.

Another notable cardiovascular effect of HAART is its impact on blood pressure. Hypertension is increasingly prevalent among PLWH on long-term HAART, partly due to metabolic changes and partly due to the direct effects of antiretroviral drugs on vascular tone and renal function. Studies suggest that the use of PIs and integrase strand transfer inhibitors (INSTIs) may be associated with an increased risk of hypertension, although the exact mechanisms remain unclear. Elevated blood pressure adds to the overall cardiovascular burden, necessitating careful monitoring and management in this population^[9,10]^.

Importantly, the cardiovascular risks vary across different ART regimens. PIs (e.g., lopinavir/ritonavir, darunavir) are generally associated with higher risks of dyslipidemia and insulin resistance, contributing significantly to cardiovascular morbidity. Certain NRTIs, such as abacavir, have also been linked to an increased risk of myocardial infarction, although this remains debated^[8]^. In contrast, tenofovir alafenamide (TAF) has a more favorable lipid profile. INSTIs, particularly dolutegravir and bictegravir, tend to have neutral or modest cardiovascular risk, although weight gain has been noted with some agents.

These differential risk profiles are increasingly informing clinical decision-making. For patients with preexisting cardiovascular disease or significant risk factors, clinicians are more inclined to avoid PIs and abacavir-containing regimens, instead opting for INSTI-based combinations with TAF or lamivudine. The shift toward more cardiometabolically favorable regimens reflects a broader movement toward personalized ART selection that integrates virologic efficacy and long-term cardiovascular safety^[17]^.

While the cardiovascular risks associated with HAART are concerning, it is important to recognize the heterogeneity of these effects across different antiretroviral drugs. For example, newer antiretroviral agents, such as dolutegravir (DTG) and tenofovir alafenamide (TAF), have improved metabolic profiles compared to older drugs. These advancements highlight the ongoing efforts to develop antiretroviral regimens that balance virologic efficacy with minimal cardiovascular toxicity. Furthermore, switching from older regimens to newer ones has been shown to reverse some of the metabolic derangements, offering a potential strategy for reducing cardiovascular risk in PLWH^[11,12]^.

In addition to drug-specific effects, the duration of HAART also influences cardiovascular outcomes. Long-term HAART use is associated with cumulative exposure to metabolic stressors, which may amplify cardiovascular risk over time. However, delaying HAART initiation to minimize cardiovascular risk is not a viable option, as untreated HIV poses an even greater threat to cardiovascular health through chronic inflammation and opportunistic infections. This underscores the importance of early HAART initiation, regular monitoring, and timely management of cardiovascular risk factors^[13,14]^.

The role of lifestyle factors in mitigating HAART-associated cardiovascular risks cannot be overstated. Smoking cessation, dietary modifications, regular physical activity, and weight management are essential components of a comprehensive cardiovascular care plan for PLWH. Additionally, pharmacologic interventions such as statins, antihypertensives, and antidiabetic drugs are crucial in managing HAART-induced metabolic derangements. For instance, atorvastatin or rosuvastatin has been shown to effectively reduce LDL-C levels in PLWH on HAART, thereby lowering the risk of atherosclerotic cardiovascular disease^[15,16]^. Interdisciplinary care models involving collaboration between infectious disease specialists, cardiologists, and primary care providers are pivotal in optimizing cardiovascular health in PLWH. These models facilitate the integration of routine cardiovascular screening into HIV care, enabling early detection and management of cardiovascular risk factors. Furthermore, patient education on the potential cardiovascular effects of HAART and the importance of adherence to prescribed therapies is vital for achieving favorable outcomes^[29,30]^.

Despite significant progress, several challenges and gaps in knowledge remain. The mechanisms underlying the cardiovascular effects of newer antiretroviral agents are not fully understood, necessitating further research. Additionally, the interplay between traditional cardiovascular risk factors (age, gender, and genetic predisposition) and HAART-associated risks requires deeper exploration. Addressing these gaps will inform the development of personalized treatment strategies that minimize cardiovascular risk while maintaining optimal virologic control^[17,31]^.

### Impact of coinfections on cardiovascular health

Hepatitis B and hepatitis C are prevalent among HIV-positive individuals due to shared modes of transmission, such as unprotected sexual contact and injection drug use. Both HBV and HCV are known to cause systemic inflammation and hepatic dysfunction, which can amplify cardiovascular risk. Chronic HCV infection, for instance, is associated with an increased risk of atherosclerosis and coronary artery disease (CAD), likely mediated by persistent immune activation and elevated levels of proinflammatory cytokines like tumor necrosis factor-alpha (TNF-α) and interleukin-6 (IL-6)^[1,2]^. HCV infection is also linked to metabolic alterations, such as insulin resistance and dyslipidemia, which are well-established risk factors for cardiovascular disease (CVD). Similarly, HBV may contribute to endothelial dysfunction and vascular damage, although its cardiovascular effects are less extensively studied than those of HCV. The presence of HIV further exacerbates these risks by intensifying immune activation and promoting a proinflammatory state. For example, coinfection with HIV and HCV has been shown to accelerate liver fibrosis and increase systemic inflammation, thereby compounding the risk of cardiovascular events^[3,4]^. This relationship underscores the need for early detection and management of HCV in HIV-positive populations, as successful antiviral treatment for HCV can significantly reduce inflammation and improve cardiovascular outcomes. However, the initiation of HAART in such patients requires careful consideration of potential drug-drug interactions, particularly with direct-acting antivirals (DAAs) used to treat HCV. Tuberculosis, another common coinfection in HIV patients, adds a unique dimension to cardiovascular risk. TB is associated with granulomatous inflammation, which can involve the cardiovascular system, leading to conditions such as pericarditis and pulmonary hypertension. HIV-associated immune suppression exacerbates the progression of TB and its cardiovascular complications, as evidenced by a higher prevalence of TB-related pericardial effusion and constrictive pericarditis in HIV-positive individuals^[5,6]^. Additionally, the chronic inflammatory response to TB can accelerate atherogenesis and contribute to myocardial dysfunction. The overlap between HIV, TB, and cardiovascular disease is particularly concerning in resource-limited settings, where access to comprehensive care may be limited. HAART, while transformative in reducing HIV-related morbidity and mortality, introduces another layer of complexity in the context of coinfections and cardiovascular health. Certain antiretroviral drugs, such as protease inhibitors, are associated with metabolic disturbances that can exacerbate CVD risk. For patients with HIV/TB coinfection, the use of rifampin-based TB therapy complicates HAART regimens due to significant drug-drug interactions. These necessary modifications may impact both HIV viral suppression and cardiovascular outcomes^[7]^. Similarly, in the context of HIV/HCV coinfection, HAART-associated hepatotoxicity can interact with HCV-related liver damage, further increasing cardiovascular risk through mechanisms such as impaired lipid metabolism and heightened systemic inflammation. Malaria, caused by Plasmodium species, is another infection with notable cardiovascular implications. Severe malaria is associated with myocardial dysfunction, arrhythmias, and metabolic acidosis, which can acutely stress the cardiovascular system^[8]^. Chronic malaria exposure has been linked to long-term cardiovascular changes, including left ventricular hypertrophy and pulmonary hypertension^[9]^. These effects are thought to be mediated by repeated systemic inflammation, endothelial activation, and microvascular damage. In regions where malaria is endemic, the coexistence of malaria and HIV poses additional challenges, as HIV impairs the immune response to malaria and may exacerbate its cardiovascular effects^[10]^. Opportunistic infections, particularly in immunocompromised individuals such as those with advanced HIV or organ transplant recipients, further complicate cardiovascular health. Pathogens such as cytomegalovirus (CMV), Epstein-Barr virus (EBV), and *Toxoplasma gondii* have been implicated in cardiovascular complications ranging from myocarditis to accelerated atherosclerosis^[11]^. CMV, for example, has been shown to induce chronic inflammation and immune activation, contributing to the development of CAD and heart failure^[12]^. Similarly, *Toxoplasma gondii* infection can cause myocarditis, particularly in individuals with weakened immune systems, and has been associated with sudden cardiac death in severe cases^[13]^. The impact of coinfections on cardiovascular health is not limited to the direct effects of pathogens and treatment regimens but also extends to social and behavioral factors. Many individuals with HIV and coinfections face barriers to healthcare access, stigma, and socioeconomic challenges, which may delay diagnosis and treatment. Furthermore, lifestyle factors such as smoking, alcohol use, and poor diet are prevalent in these populations and contribute to the overall burden of cardiovascular disease^[8]^.

### Role of inflammation and immune activation

HIV-induced inflammation initiates and accelerates atherosclerotic processes through multiple mechanisms. Chronic activation of the immune system results in the production of proinflammatory cytokines, such as interleukin-6 (IL-6), tumor necrosis factor-alpha (TNF-α), and C-reactive protein (CRP), which are central to the inflammatory cascade associated with endothelial dysfunction and vascular damage. These cytokines promote monocyte activation and recruitment to the vascular endothelium, where they differentiate into macrophages and uptake oxidized low-density lipoproteins (LDL) to form foam cells – key components of atherosclerotic plaques^[3,4]^. Moreover, endothelial dysfunction, a hallmark of atherosclerosis, is exacerbated in HIV infection due to the direct effects of viral proteins like gp120 and Tat, which disrupt endothelial integrity and increase the permeability of vascular walls. This dysfunction is compounded by oxidative stress, a byproduct of chronic immune activation, which further damages endothelial cells and promotes plaque formation^[5]^. Studies have demonstrated that HIV-positive individuals exhibit higher levels of arterial inflammation and subclinical atherosclerosis, as evidenced by increased carotid intima-media thickness and coronary artery calcium scores compared to HIV-negative individuals^[6,7]^. Beyond atherosclerosis, chronic immune activation in HIV infection contributes to myocardial dysfunction through pathways involving both direct viral effects and systemic inflammation. Persistent activation of CD4+ and CD8+ T cells, as well as monocyte and macrophage activation, results in the release of inflammatory mediators such as interferon-gamma (IFN-γ) and TNF-α, which have cardiotoxic effects. These mediators induce apoptosis of cardiomyocytes, fibrosis, and cardiac tissue remodeling, ultimately impairing cardiac function^[8,9]^. In addition to systemic inflammation, the translocation of microbial products such as lipopolysaccharides (LPS) from the gut into the bloodstream due to HIV-associated gut epithelial barrier dysfunction exacerbates immune activation. Elevated levels of LPS and other microbial antigens have been strongly associated with myocardial inflammation and fibrosis in HIV-positive individuals^[10]^. This mechanism is thought to underlie the increased prevalence of HIV-associated cardiomyopathy, which is characterized by left ventricular dysfunction and dilatation.

Several biomarkers have been identified to quantify the extent of inflammation and immune activation in individuals with HIV and to predict cardiovascular risk. IL-6 is one of the most extensively studied proinflammatory cytokines in this context. Elevated IL-6 levels have been correlated with increased carotid artery stiffness, a surrogate marker of subclinical atherosclerosis, and a higher incidence of major adverse cardiovascular events (MACE) in HIV-positive populations^[11,12]^. Similarly, CRP, an acute-phase reactant, is a well-established marker of systemic inflammation. High-sensitivity CRP (hs-CRP) assays have revealed elevated levels in HIV-positive individuals, which are independently associated with endothelial dysfunction and coronary artery disease^[13]^. Another critical biomarker is D-dimer, a fibrin degradation product that reflects ongoing coagulation and fibrinolysis. Elevated D-dimer levels have been linked to hypercoagulability, increased arterial thrombosis, and a higher risk of myocardial infarction in individuals with HIV^[14,15]^. Additional biomarkers, including soluble CD14 (sCD14) and soluble CD163 (sCD163), reflect monocyte and macrophage activation, respectively, and have been implicated in the pathogenesis of atherosclerosis and myocardial dysfunction.

Studies have shown that higher levels of sCD14 and sCD163 are associated with greater carotid plaque burden and left ventricular hypertrophy in HIV-positive cohorts^[16,29]^. While ART effectively suppresses HIV replication and reduces immune activation, it does not completely normalize inflammation. Persistent inflammation in ART-treated individuals is multifactorial, involving residual viral replication, latent HIV reservoirs, and ART-related metabolic side effects. For instance, protease inhibitors, a class of ART, are associated with dyslipidemia and insulin resistance, which exacerbate systemic inflammation and contribute to cardiovascular risk^[30]^. Despite these challenges, newer ART regimens with improved safety profiles are being developed to minimize inflammation. For example, integrase strand transfer inhibitors (INSTIs) have shown promise in reducing inflammatory markers such as IL-6 and D-dimer compared to older ART regimens^[31]^. However, long-term data on their cardiovascular effects are still emerging.

### Clinical presentation and diagnosis

**Figure 3.**
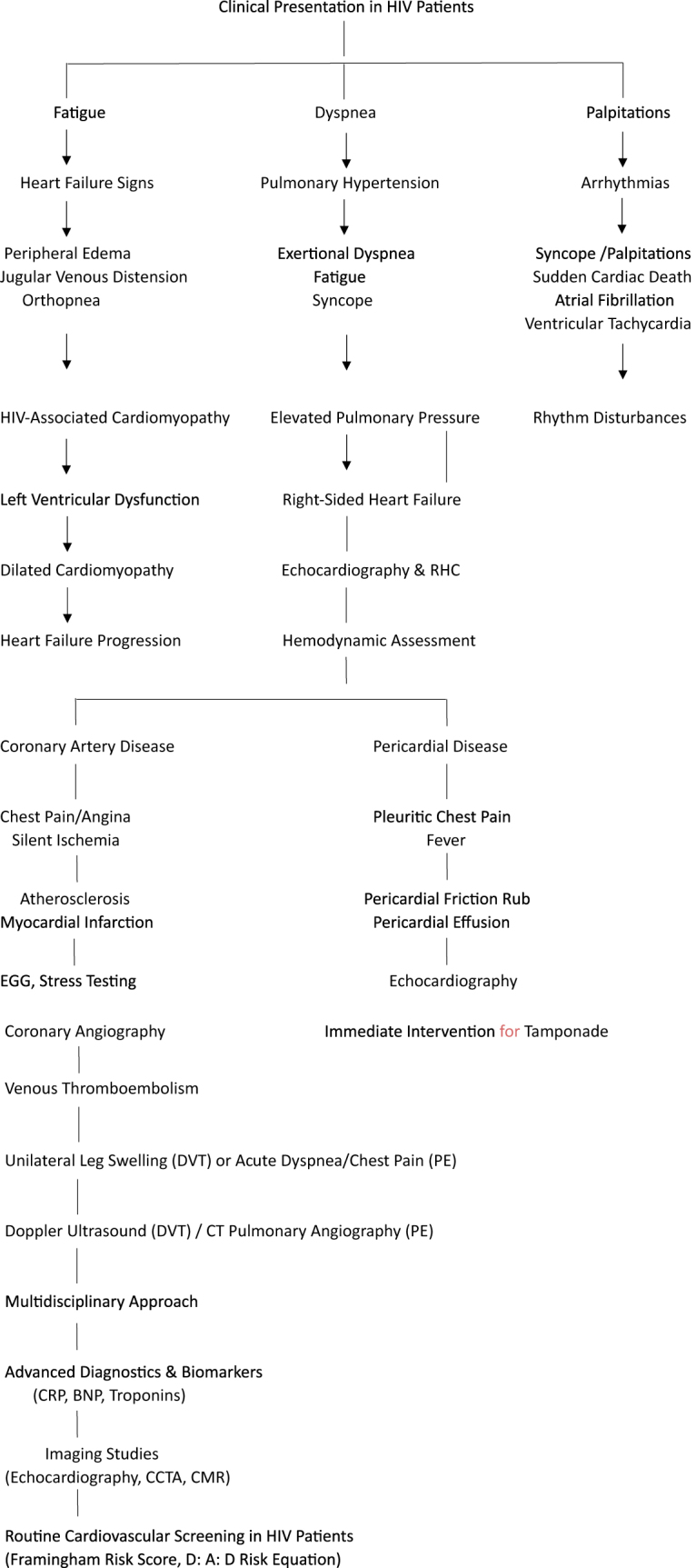
Impact of HAART on Lipid Profiles and Insulin Sensitivity.

Patients with HIV may present with nonspecific symptoms such as fatigue, dyspnea, and palpitations, which can complicate the differentiation of cardiac disease from other HIV-related conditions like anemia or pulmonary infections (see Fig. 3, which presents a flowchart integrating symptoms, associated complications, and diagnostic strategies for cardiovascular manifestations in HIV patients to guide clinical evaluation). In more advanced cases, individuals might exhibit signs of overt heart failure, including peripheral edema, jugular venous distension, and orthopnea. These symptoms often result from HIV-associated cardiomyopathy, which is a recognized complication in untreated or late-stage HIV infection. Cardiomyopathy in this context is often dilated, characterized by impaired left ventricular systolic function, and may progress to severe heart failure if not addressed^[3,4]^. Another frequent cardiovascular complication in HIV patients is pericardial disease. Pericardial effusion, often associated with opportunistic infections like tuberculosis or fungal diseases, is a common presentation. Patients may complain of pleuritic chest pain, fever, and a pericardial friction rub on auscultation. In severe cases, cardiac tamponade can occur, requiring immediate intervention. Echocardiography is the diagnostic modality for identifying pericardial effusion and assessing hemodynamic compromise^[5,6]^. Coronary artery disease (CAD) has become increasingly prevalent in individuals with HIV, particularly those on long-term HAART. Chronic systemic inflammation and immune activation, alongside traditional risk factors like smoking, dyslipidemia, and hypertension, contribute to an accelerated process of atherosclerosis. Patients may present with symptoms of angina or, in acute cases, myocardial infarction. Silent ischemia is also common, emphasizing this population’s need for regular cardiovascular screening. Electrocardiography (ECG) and stress testing are often used as initial diagnostic tools, while coronary angiography remains the gold standard for confirming the diagnosis and planning interventions^[7,8]^. Pulmonary hypertension is another significant cardiovascular manifestation in HIV patients. The exact pathophysiology is multifactorial, involving direct viral effects, chronic inflammation, and increased susceptibility to thromboembolic events. Patients typically present with exertional dyspnea, fatigue, and syncope, which may progress to right-sided heart failure. Diagnostic evaluation includes transthoracic echocardiography to estimate pulmonary artery pressures, followed by right heart catheterization for definitive diagnosis and hemodynamic assessment. Pulmonary function tests and imaging studies are also useful for excluding other potential causes of dyspnea^[9,10]^. Arrhythmias, including atrial fibrillation and ventricular tachycardia, are increasingly recognized among HIV patients. These rhythm disturbances are often secondary to structural heart disease, drug toxicity, or electrolyte imbalances. Patients may present with palpitations, syncope, or even sudden cardiac death. Holter monitoring and electrophysiological studies are valuable diagnostic tools for assessing arrhythmias and guiding treatment decisions. Medications used in HAART regimens, such as protease inhibitors, have been implicated in QT prolongation, necessitating regular ECG monitoring for early detection of life-threatening arrhythmias like Torsades de Pointes^[11,12]^. In addition to these manifestations, HIV patients are at an increased risk for venous thromboembolism (VTE), including deep vein thrombosis and pulmonary embolism. Chronic inflammation, endothelial dysfunction, and hypercoagulability associated with HIV infection contribute to this heightened risk. Clinically, patients may present with unilateral leg swelling, pain, and tenderness in cases of deep vein thrombosis or acute onset of chest pain, dyspnea, and hypoxemia in pulmonary embolism. Doppler ultrasound is the diagnostic modality of choice for suspected deep vein thrombosis, while computed tomography pulmonary angiography is the gold standard for confirming pulmonary embolism^[13,14]^. The diagnosis of cardiovascular complications in HIV patients requires a multidisciplinary approach that combines clinical evaluation with advanced diagnostic tools. Biomarkers such as C-reactive protein (CRP), brain natriuretic peptide (BNP), and cardiac troponins have emerged as valuable adjuncts in assessing cardiovascular risk and detecting subclinical disease. Elevated CRP levels reflect underlying inflammation and are associated with an increased risk of myocardial infarction and other adverse cardiovascular events. Similarly, BNP is useful for diagnosing heart failure, particularly in distinguishing cardiac from noncardiac causes of dyspnea in HIV patients. Troponins are essential for diagnosing acute coronary syndromes, as elevated levels indicate myocardial injury, whether due to ischemic or nonischemic causes^[15,16]^. Imaging studies play a pivotal role in the diagnosis of cardiovascular disease in HIV patients. Transthoracic echocardiography is widely used to evaluate structural heart abnormalities, assess ventricular function, and detect pericardial effusion. Noninvasive imaging modalities such as coronary computed tomography angiography (CCTA) and cardiac magnetic resonance imaging (CMR) in suspected coronary artery disease cases provide detailed anatomical and functional information. CMR is particularly useful for differentiating ischemic from nonischemic cardiomyopathies and assessing myocardial fibrosis^[29,30]^. The role of routine cardiovascular screening in asymptomatic HIV patients is increasingly emphasized, given the high prevalence of subclinical disease. Guidelines recommend periodic assessment of traditional cardiovascular risk factors, such as blood pressure, lipid profiles, and glucose levels, in addition to HIV-specific factors like CD4 count and viral load. Risk stratification tools, such as the Framingham Risk Score and the D:A:D (Data Collection on Adverse Effects of Anti-HIV Drugs) study risk equation, can help identify high-risk individuals who may benefit from early intervention^[17,31]^.

This flowchart integrates the symptoms, associated complications, and diagnostic strategies for cardiovascular manifestations in HIV patients, ensuring a clear pathway for clinical evaluation – Source: Authors’ Creation.

### Management and treatment strategies

A cornerstone of management is addressing modifiable cardiovascular risk factors, which are prevalent in people living with HIV (PLWH). These include smoking, hypertension, hyperlipidemia, obesity, and sedentary lifestyles. Smoking cessation programs tailored for PLWH have shown promise in reducing cardiovascular events, particularly as smoking is more prevalent among HIV-positive populations compared to the general population^[28]^. Similarly, dietary and exercise interventions targeting obesity and metabolic syndrome are crucial, given the heightened risk of insulin resistance and dyslipidemia associated with HAART^[32]^.

Pharmacologic interventions play a pivotal role in managing the cardiovascular health of PLWH. Statins are commonly employed to treat dyslipidemia, a frequent side effect of protease inhibitors (PIs) and some non-nucleoside reverse transcriptase inhibitors (NNRTIs) (see Table 1). However, drug-drug interactions between statins and antiretrovirals require careful selection of agents. For example, atorvastatin and simvastatin are contraindicated with PIs due to the risk of myopathy, while pravastatin and pitavastatin are safer alternatives^[33]^. Additionally, antihypertensive therapy is critical for managing the increased prevalence of hypertension in HIV-positive individuals, with angiotensin-converting enzyme (ACE) inhibitors and calcium channel blockers being commonly used^[34]^.

Adjusting HAART regimens is often necessary to mitigate cardiovascular risks. Although HAART has revolutionized HIV management by reducing viral loads and improving survival, certain antiretroviral drugs are associated with adverse cardiovascular effects. For instance, abacavir has been linked to an increased risk of myocardial infarction, particularly in individuals with high cardiovascular risk profiles^[35]^. Switching to alternative agents, such as tenofovir, which has a more favorable lipid profile, can reduce these risks^[36]^. Close monitoring and individualized therapy are essential to optimize outcomes. Lifestyle modification programs are an integral component of cardiovascular risk management in PLWH. Comprehensive counseling on diet, physical activity, and stress reduction has demonstrated effectiveness in reducing cardiovascular disease (CVD) burden^[37]^. Integrating behavioral health interventions, including cognitive behavioral therapy and peer support groups, further enhances adherence to lifestyle changes and medication regimens.

A critical component of contemporary HIV care must be the routine integration of cardiovascular screening protocols. Stronger advocacy is needed for the universal adoption of cardiovascular risk assessments as a standard of care in HIV management settings. Baseline and periodic evaluations of lipid profiles, blood pressure, glucose levels, and inflammatory markers should be incorporated systematically to facilitate the early identification of at-risk individuals. Cardiovascular risk calculators specifically adapted for PLWH – such as the D:A:D (Data Collection on Adverse Events of Anti-HIV Drugs) risk score – can aid in stratifying patients and guiding personalized interventions^[27]^. Emerging evidence underscores the importance of regular cardiovascular screening for PLWH. Routine assessment of lipid profiles, blood pressure, and glucose levels facilitates the early identification of at-risk individuals. Advanced imaging modalities, such as coronary computed tomography angiography (CCTA), can detect subclinical atherosclerosis and guide preventive measures^[38]^. Biomarkers such as high-sensitivity C-reactive protein (hs-CRP) and interleukin-6 are being explored for their utility in risk stratification and monitoring treatment efficacy^[39]^.

Equally essential is the proactive application of early cardiovascular intervention strategies within HIV clinics. Implementing structured prevention protocols – such as risk calculators tailored to PLWH, cardiovascular counseling, and timely initiation of cardioprotective therapies – can reduce long-term morbidity and mortality^[8]^

Interdisciplinary care models integrating cardiology and infectious disease expertise are critical for optimal management. These models enable comprehensive care, including the timely identification and treatment of cardiovascular complications. For example, the inclusion of cardiologists in HIV clinics has been associated with improved outcomes through coordinated care and early intervention^[40]^. Moreover, expanding access to telemedicine and mobile health applications offers new avenues for managing cardiovascular health in resource-limited settings. Prevention of cardiovascular complications begins with the early initiation of HAART. Studies have shown that starting antiretroviral therapy at higher CD4 counts reduces immune activation and inflammation, thereby decreasing cardiovascular risk^[41]^.

Additionally, novel antiretroviral agents with improved cardiovascular safety profiles are being developed, offering hope for safer long-term management^[42]^. Therapeutic strategies targeting immune activation and inflammation, such as interleukin-1 inhibitors and monoclonal antibodies, are also under investigation and may complement existing approaches^[43]^. Despite these advances, significant challenges remain in managing cardiovascular health in PLWH. Social determinants of health, including stigma, poverty, and limited access to care, exacerbate disparities in cardiovascular outcomes. Addressing these factors through targeted public health initiatives and policy changes is essential for achieving equitable care. Furthermore, ongoing research is needed to elucidate the long-term cardiovascular effects of newer antiretroviral agents and to develop innovative risk reduction strategies^[44-46]^.

### Challenges and controversies

**Figure 4.**
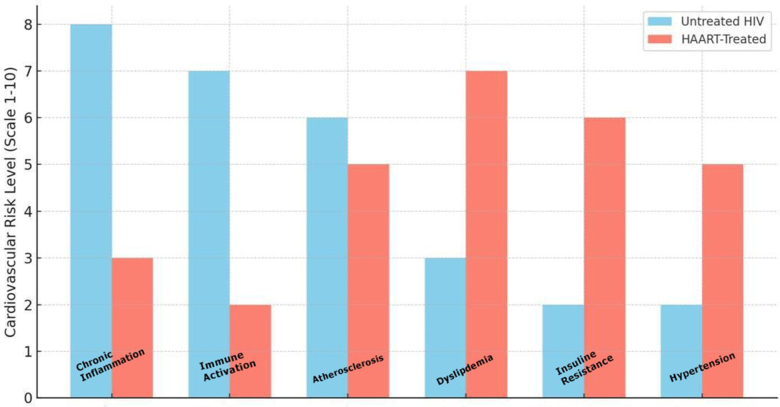
Diagnostic Pathway for Cardiovascular Manifestations in HIV Patients.

One of the primary challenges is disentangling the independent contributions of HIV infection and HAART to cardiovascular risk. Chronic HIV infection is characterized by persistent immune activation and inflammation, which are believed to accelerate atherosclerosis and increase the risk of cardiovascular disease (CVD) (see Fig. 4, which compares cardiovascular risk factors in untreated HIV versus individuals on HAART, highlighting both the reduction in inflammation and the rise in metabolic complications)^[1,2]^. Studies have shown elevated levels of proinflammatory markers such as interleukin-6 (IL-6) and C-reactive protein (CRP) in PLWH, even among those with suppressed viral loads on HAART^[3]^. These inflammatory processes contribute to endothelial dysfunction, plaque formation, and an increased likelihood of myocardial infarction and stroke. However, determining the extent to which these outcomes are directly attributable to the virus versus the effects of HAART is an ongoing challenge. HAART itself introduces a set of cardiovascular risks that complicate the management of HIV. Protease inhibitors (PIs), a cornerstone of many HAART regimens, have been linked to dyslipidemia, including elevated low-density lipoprotein (LDL) cholesterol and triglycerides, as well as reduced high-density lipoprotein (HDL) cholesterol levels^[4,5]^.

Here is a comparative bar chart illustrating cardiovascular risk factors between individuals with untreated HIV infection and those on HAART. The chart highlights the reduction in risks such as chronic inflammation and immune activation with HAART but also demonstrates the increased risks of dyslipidemia, insulin resistance, and hypertension due to antiretroviral therapy – Source: Authors’ Creation.

Additionally, integrase strand transfer inhibitors (INSTIs) have been associated with weight gain and metabolic syndrome, further increasing cardiovascular risk^[6]^. While newer antiretroviral drugs are being developed to minimize these adverse effects, the long-term safety profiles of these medications remain uncertain, particularly as the HIV-positive population ages. The aging of the HIV-positive population introduces another layer of complexity. Thanks to HAART, PLWH are living longer. Still, this extended lifespan exposes them to age-related cardiovascular risks that may be exacerbated by their HIV status and long-term antiretroviral therapy^[7]^. Older HIV-positive individuals often have higher rates of hypertension, diabetes, and dyslipidemia compared to their HIV-negative counterparts, making it difficult to isolate the effects of HIV and HAART on cardiovascular outcomes. Furthermore, traditional cardiovascular risk scores, such as the Framingham Risk Score, may underestimate the risk in this population, highlighting the need for HIV-specific risk assessment tools^[8]^.

Another controversy surrounds the lack of standardized, HIV-specific cardiovascular risk assessment tools. Existing models often underestimate risk in PLWH, underscoring the need for tools that account for HIV-related inflammation, ART exposure, and immune status. Without accurate assessment, early intervention may be delayed, compounding long-term cardiovascular morbidity^[32]^.

Adherence to HAART presents additional challenges, particularly when considering its cardiovascular implications. For some patients, weight gain or dyslipidemia concerns may lead to suboptimal adherence, increasing the risk of viral rebound and immune activation^[9]^. Balancing the benefits of viral suppression with the need to mitigate cardiovascular risk requires careful patient education and shared decision-making. This is particularly important in resource-limited settings, where access to lipid-lowering agents and other cardiovascular medications may be constrained. Another controversial area is the role of lifestyle interventions in reducing cardiovascular risk among PLWH. While lifestyle modifications, such as smoking cessation, regular physical activity, and dietary changes, are universally recommended for cardiovascular prevention, their efficacy in the context of chronic HIV infection and HAART remains underexplored^[10]^.

Smoking, in particular, is disproportionately prevalent among PLWH and is a major modifiable risk factor for CVD^[47-49]^. However, the interaction between smoking, HIV-related inflammation, and HAART-induced metabolic changes is not fully understood, making it difficult to quantify the benefits of cessation in this population. The impact of socioeconomic and structural factors cannot be overlooked when discussing cardiovascular health in PLWH. In many settings, HIV disproportionately affects marginalized populations, including racial and ethnic minorities, people who use drugs, and individuals with low socioeconomic status^[12]^. These groups often face barriers to accessing high-quality healthcare, including cardiovascular screening and management. Additionally, stigma associated with HIV can discourage individuals from seeking care, further compounding health disparities.

**Figure 5.**
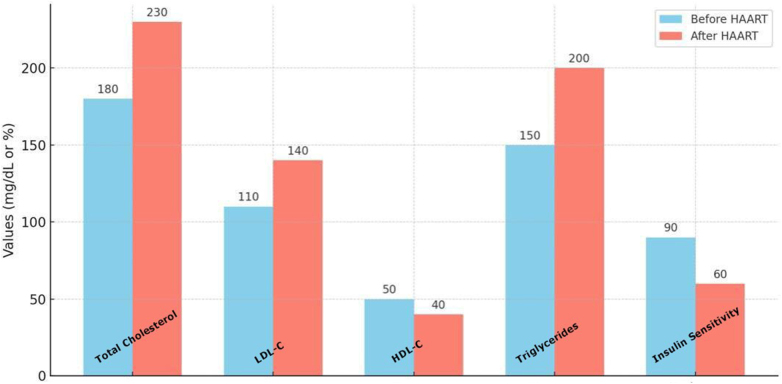
Comparative Analysis of Cardiovascular Risk Factors in Untreated HIV and HAART-Treated Patients.

Addressing these structural inequities is essential for improving cardiovascular outcomes but requires a multidisciplinary approach beyond the clinical setting. A key challenge lies in the inconsistent implementation of cardiovascular screening in HIV care. Despite guidelines emphasizing its importance, many HIV treatment centers lack the infrastructure or clinical protocols to assess cardiovascular risk routinely. This results in missed opportunities for early detection and intervention. Early initiation of HAART has been shown to reduce HIV-related inflammation and immune activation, which may lower the risk of cardiovascular complications^[13]^. However, initiating early treatment exposes patients to the potential long-term adverse effects of antiretroviral drugs, including their impact on lipid profiles and glucose metabolism (see Fig. 5, which illustrates changes in lipid profiles – total cholesterol, LDL-C, HDL-C, and triglycerides – and insulin sensitivity before and after HAART initiation, particularly with protease inhibitors). Balancing these considerations is particularly challenging in patients with preexisting cardiovascular risk factors, such as obesity or a family history of CVD. Pharmacological management of cardiovascular risk in PLWH is another contentious area.

This bar graph illustrates the changes in lipid profiles (total cholesterol, LDL-C, HDL-C, and triglycerides) and insulin sensitivity before and after HAART initiation, particularly with protease inhibitors (PIs) – source: Authors’ Creation.

While statins are widely used to manage dyslipidemia, some evidence suggests that they may have differential efficacy and safety profiles in HIV-positive individuals compared to the general population^[14]^. For example, drug-drug interactions between statins and certain antiretroviral agents, such as ritonavir-boosted PIs, can increase the risk of adverse effects, including myopathy and liver dysfunction^[15]^. Additionally, the potential anti-inflammatory effects of statins in the context of HIV are not well understood, raising questions about their utility beyond lipid-lowering.

There is also an ongoing debate about when and how to initiate cardiovascular preventive therapies, such as statins or antihypertensives, in asymptomatic PLWH with borderline risk. These gray areas highlight the need for more targeted guidelines and greater emphasis on cardiovascular prevention within HIV treatment paradigms^[1]^.

Research gaps further complicate the understanding and management of cardiovascular risk in HIV. Most studies to date have been conducted in high-income countries, with limited data available from low- and middle-income countries, where the burden of HIV is highest^[16]^. These settings often face unique challenges, including a higher prevalence of untreated hypertension and diabetes, which can exacerbate the cardiovascular effects of HIV and HAART. Additionally, the lack of long-term cohort studies makes it difficult to assess the lifetime cardiovascular risk associated with chronic HIV infection and antiretroviral therapy.

Finally, greater collaboration between cardiologists and infectious disease specialists is needed to manage PLWH with cardiovascular complications. Historically, the focus of HIV care has been on virological suppression, with less emphasis on comorbidities such as CVD. However, as the population of HIV-positive individuals continues to age, integrating cardiovascular care into routine HIV management will be essential. This requires interdisciplinary collaboration and the development of clinical guidelines that address the unique cardiovascular risks PLWH face^[50-52]^.

### Research gaps and future directions

One of the major research gaps is the incomplete understanding of the exact mechanisms through which HIV independently predisposes patients to cardiovascular diseases (CVD). Chronic inflammation, immune activation, and direct viral effects on cardiovascular tissues have been implicated, but these mechanisms are not fully elucidated. The precise contribution of each of these factors, especially in the context of controlled HIV infection under HAART, remains ambiguous. Studies utilizing advanced molecular and cellular techniques, including single-cell RNA sequencing and CRISPR gene editing, could illuminate the pathways involved^[53-55]^. Additionally, there is limited research on how these mechanisms interact with traditional cardiovascular risk factors, such as hypertension and diabetes, in HIV-positive individuals. The role of HAART in contributing to cardiovascular risk is another area where significant gaps exist. While HAART has transformed HIV from a fatal disease into a manageable chronic condition, it has introduced new challenges in the form of metabolic complications. Protease inhibitors and some nucleoside reverse transcriptase inhibitors have been linked to dyslipidemia, insulin resistance, and accelerated atherosclerosis^[56]^. However, the differential cardiovascular effects of various HAART regimens are not well-documented, particularly in diverse populations. Large-scale, multicenter longitudinal studies are needed to determine newer antiretroviral agents’ long-term cardiovascular safety profiles. Moreover, pharmacogenomics research could help identify genetic predispositions to HAART-induced cardiovascular side effects, enabling personalized treatment approaches. Another critical gap is the lack of data on the cardiovascular health of women living with HIV. Most cardiovascular research on HIV has predominantly focused on male populations, leading to a dearth of knowledge about gender-specific risks and outcomes. Women may have different cardiovascular risk profiles due to hormonal differences, unique immunological responses, and interactions with HAART^[57]^. Further studies focusing on women are crucial to developing gender-sensitive cardiovascular care strategies. Similarly, there is insufficient research on the cardiovascular health of aging HIV-positive populations. With improved life expectancy due to HAART, a growing proportion of people living with HIV are older adults. Aging is independently associated with increased cardiovascular risk, and its interplay with HIV and HAART is poorly understood. Research on the combined impact of aging, HIV, and HAART on cardiovascular health could inform strategies to address the unique needs of this demographic^[58,59]^. Investigations into biomarkers of cardiovascular aging, such as telomere length and epigenetic modifications, could provide valuable insights. The limited data availability from low- and middle-income countries (LMICs) represents another significant gap. Most studies on HIV and cardiovascular health have been conducted in high-income countries, where the epidemiological profiles of both HIV and CVD differ substantially from those in LMICs. Factors such as coinfections (e.g., tuberculosis and hepatitis), malnutrition, and limited access to healthcare may modulate the cardiovascular impact of HIV and HAART in LMICs^[7]^. Therefore, region-specific research must address these disparities and develop contextually relevant interventions. Prevention strategies for cardiovascular complications in HIV-positive individuals also require further investigation. Although lifestyle interventions and routine cardiovascular screening are recommended, their effectiveness in this population has not been rigorously evaluated. Randomized controlled trials examining the impact of structured exercise programs, dietary modifications, and smoking cessation initiatives on cardiovascular outcomes in HIV-positive patients would provide valuable evidence^[8]^. Moreover, the potential role of adjunctive therapies, such as anti-inflammatory agents or immune modulators, in reducing cardiovascular risk warrants exploration. The development of safer antiretroviral drugs is a critical area for future research. While newer agents, such as integrase strand transfer inhibitors (INSTIs), have improved safety profiles, concerns about their long-term cardiovascular effects persist. Investigating the molecular mechanisms underlying these effects could guide the design of antiretroviral agents with minimal cardiovascular toxicity. Furthermore, research into alternative drug delivery systems, such as long-acting injectables and nanotechnology-based formulations, could improve adherence while reducing systemic side effects^[9]^. Understanding the burden of cardiovascular disease in pediatric and adolescent HIV populations is another area requiring attention. Children and adolescents living with HIV, whether infected perinatally or through other routes, represent a unique cohort with distinct cardiovascular risks. The long-term effects of early HAART initiation on cardiovascular development are not well-documented. Longitudinal studies following these populations into adulthood are essential to elucidate the lifelong cardiovascular consequences of HIV and its treatment^[10]^. Finally, there is a need for innovative research approaches to address the complexities of HIV and cardiovascular health. Multidisciplinary collaborations involving cardiologists, infectious disease specialists, immunologists, and epidemiologists could facilitate comprehensive investigations. The integration of artificial intelligence (AI) and machine learning in data analysis could uncover patterns and predictors of cardiovascular risk in HIV-positive populations. Additionally, establishing large, global consortia to pool data from diverse cohorts would enhance the generalizability of research findings^[60-62]^.

## Concluding remarks

HIV and its treatment with HAART have profound and multifaceted effects on cardiovascular health. While HAART has significantly improved the prognosis and life expectancy of individuals living with HIV, its long-term use is associated with metabolic disturbances and cardiovascular complications. Chronic inflammation and immune activation caused by the virus further exacerbate these risks, contributing to conditions such as atherosclerosis, cardiomyopathy, and coronary artery disease. Effective management of cardiovascular complications in HIV patients requires a multidisciplinary approach that integrates regular cardiovascular monitoring, lifestyle modifications, and optimization of HAART regimens to minimize adverse effects. Additionally, early intervention and prevention strategies, including smoking cessation, weight management, and early initiation of treatment, are critical for reducing cardiovascular risks in this population. Future research should focus on understanding the mechanisms underlying these cardiovascular effects and developing safer antiretroviral therapies. By addressing these gaps, healthcare providers can better balance HIV management with cardiovascular health, ultimately improving the overall quality of life for individuals living with HIV.

### Call to action

Healthcare providers, researchers, and policymakers must prioritize the integration of cardiovascular health into the comprehensive care of individuals living with HIV. This includes the mandatory implementation of regular cardiovascular screening protocols as a routine aspect of HIV management – particularly for patients on long-term HAART or those with existing risk factors.

Clinicians are encouraged to adopt a multidisciplinary approach that includes cardiologists and other specialists. Early identification of at-risk individuals and prompt initiation of evidence-based cardiovascular interventions must be embedded into HIV care pathways. Expanding training and infrastructure for cardiovascular risk assessment in HIV clinics, particularly in low- and middle-income countries, should also be a global priority.

Future research must aim to develop HIV-specific cardiovascular risk tools, validate early intervention models, and evaluate the long-term outcomes of integrated care strategies. Only through a comprehensive and proactive approach can we effectively reduce the cardiovascular burden in people living with HIV.

## Data Availability

The views and opinions expressed in this paper are solely those of the author and do not necessarily reflect the official policies or positions of any affiliated institution or organization. The author declares no conflicts of interest or financial relationships relevant to this research. All data generated or analyzed during this study are included in this published article and its supplementary information files.

## References

[1] Vos AG Venter WDF. Cardiovascular toxicity of contemporary antiretroviral therapy. Curr Opin HIV AIDS 2021;16:286–291. 34545036 10.1097/COH.0000000000000702

[2] Chen GJ Sun HY Chang SY. Incidence and impact of low-level viremia among people living with HIV who received protease inhibitor- or dolutegravir-based antiretroviral therapy. Int J Infect Dis 2021;105:147–151. 33592339 10.1016/j.ijid.2021.02.045

[3] Lagathu C Béréziat V Gorwood J. Metabolic complications affecting adipose tissue, lipid, and glucose metabolism associated with HIV antiretroviral treatment. Expert Opin Drug Saf 2019;18:829–840. 31304808 10.1080/14740338.2019.1644317

[4] Cai Q Pan W Zhang C. The relationship between HIV/AIDS and coronary heart disease: a bibliometric analysis. Medicine (Baltimore) 2024;103:e39831. 39465717 10.1097/MD.0000000000039831 PMC11460847

[5] Martini S Pisaturo M Russo A. Evaluation of lipid profile and intima-media thickness in antiretroviral-experienced HIV-infected patients treated with protease inhibitor-based regimens versus protease inhibitor-sparing regimens. Pathogens 2023;12:925. 37513772 10.3390/pathogens12070925 PMC10383365

[6] Jameson JL Fauci AS Kasper DL Harrison’s principles of internal medicine. 21st ed. McGraw Hill; 2022. https://accessmedicine.mhmedical.com/book.aspx?bookID=3095

[7] Zipes DP Libby P Bonow RO. Braunwald’s heart disease: a textbook of cardiovascular medicine. 12th ed. Elsevier; 2021. https://www.clinicalkey.com/#!/content/book/3-s2.0-B9780323722193000852

[8] Bennett JE Dolin R Mandell GL, Douglas RG, and Bennett’s principles and practice of infectious diseases. 9th ed. Elsevier; 2020. https://shop.elsevier.com/books/mandell-douglas-and-bennetts-principles-and-practice-of-infectious-diseases/bennett/978-0-323-48255-4

[9] Kaplan RC Hanna DB Kizer JR. Recent insights into cardiovascular disease (CVD) risk among HIV-infected adults. HIV/AIDS Rep 2016; 13:44–52. 10.1007/s11904-016-0301-4 PMC6336192 26910597

[10] So-Armah K Freiberg MS. HIV and cardiovascular disease: update on clinical events, special populations, and novel biomarkers. Curr HIV/AIDS Rep 2018;15:233–244. 29752699 10.1007/s11904-018-0400-5 PMC6230511

[11] Vos AG Hoeve K Barth RE. Cardiovascular disease risk in an urban African population: a cross-sectional analysis on the role of HIV and antiretroviral treatment. Retrovirology 2019;16:37. 31796103 10.1186/s12977-019-0497-7 PMC6889610

[12] Knobel H Domingo P Suárez-Lozano I. Rate of cardiovascular, renal, and bone disease and their major risk factors in HIV-infected individuals on antiretroviral therapy in Spain. Enferm Infecc Microbiol Clin (Engl Ed) 2019;37:373–379. 30389268 10.1016/j.eimc.2018.09.015

[13] Shah AS Stelzle D Lee KK. Global burden of atherosclerotic cardiovascular disease in people living with HIV: systematic review and meta-analysis. Circulation 2018;138:1100–1112. 29967196 10.1161/CIRCULATIONAHA.117.033369 PMC6221183

[14] Zareba KM Miller TL Lipshultz SE. Cardiovascular disease and toxicities related to HIV infection and its therapies. Expert Opin Drug Saf 2005;4:1017–1025. 16255661 10.1517/14740338.4.6.1017

[15] Lipshultz SE Mas CM Henkel JM. HAART to heart: highly active antiretroviral therapy and the risk of cardiovascular disease in HIV-infected or exposed children and adults. Expert Rev Anti Infect Ther 2012;10:661–674. 22734956 10.1586/eri.12.53

[16] Calza L Manfredi R Pocaterra D. Risk of premature atherosclerosis and ischemic heart disease associated with HIV infection and antiretroviral therapy. J Infect 2008;57:16–32. 18358535 10.1016/j.jinf.2008.02.006

[17] Zhang Y Xiao J Zhang W. Cumulative effects of hypertriglyceridemia in HIV-infected patients switching from NNRTIs to PI-based antiretroviral therapy. J Infect Dev Ctries 2022;16:528–536. 35404860 10.3855/jidc.12519

[18] Byonanebye DM Polizzotto MN Law M. Incidence of dyslipidemia in people with HIV who are treated with integrase inhibitors versus other antiretroviral agents. AIDS 2021;35:82. 10.1097/QAD.0000000000002811 33443370

[19] O’Halloran JA Sahrmann J Butler AM. Brief report: integrase strand transfer inhibitors are associated with a lower risk of incident cardiovascular disease in people living with HIV. J Acquir Immune Defic Syndr 2020;84:396–399. 32243280 10.1097/QAI.0000000000002357 PMC7401319

[20] Bakal DR Coelho LE Luz PM. Obesity following ART initiation is common and influenced by both traditional and HIV-/ART-specific risk factors. J Antimicrob Chemother 2018;73:2177–2185. 29722811 10.1093/jac/dky145 PMC6054231

[21] Hamooya BM Mulenga LB Masenga SK. Metabolic syndrome in Zambian adults with human immunodeficiency virus on antiretroviral therapy: prevalence and associated factors. Medicine (Baltimore) 2021;100:e00000. 10.1097/MD.0000000000025236 PMC8036111 33832083

[22] Tiarukkitsagul J Sungkanuparph S. Assessment of atherosclerotic cardiovascular disease risks among people living with HIV receiving first-line and second-line antiretroviral therapy in a resource-limited setting. Tiarukkitsagul J, Sungkanuparph S Int J STD AIDS 2021;32:421–426. 33533302 10.1177/0956462420972855

[23] Serrão R Piñero C Vélez J. Non-AIDS-related comorbidities in people living with HIV-1 aged 50 years and older: the AGING POSITIVE study. Int J Infect Dis 2019;79:94–100. 30529370 10.1016/j.ijid.2018.10.011

[24] Bastard JP Couffignal C Fellahi S. Diabetes and dyslipidemia are associated with oxidative stress independently of inflammation in long-term antiretroviral-treated HIV-infected patients. Diabetes Metab 2019;45:573–81. 30862472 10.1016/j.diabet.2019.02.008

[25] Agu CE Uchendu IK Nsonwu AC. Prevalence and associated risk factors of peripheral artery disease in virologically suppressed HIV-infected individuals on antiretroviral therapy in Kwara state, Nigeria: a cross-sectional study. BMC Public Health 2019;19:1143. 31429736 10.1186/s12889-019-7496-4 PMC6700806

[26] Calza L Borderi M Granozzi B. Vitamin D insufficiency is associated with subclinical atherosclerosis in HIV-1-infected patients on combination antiretroviral therapy. HIV Res Clin Pract 2019;20:131–139. 32065065 10.1080/25787489.2020.1724749

[27] Alikhani A Morin H Matte S. Association between lipodystrophy and length of exposure to ARTs in adult HIV-1-infected patients in montreal. BMC Infect Dis 2019;19:820. 31533648 10.1186/s12879-019-4446-9 PMC6751890

[28] Llibre JM López-Cortés LF Aylott A. Brief report: evaluation of inflammation and atherogenesis biomarkers through 148 weeks postswitch to dolutegravir and rilpivirine in SWORD-1/SWORD-2. J Acquir Immune Defic Syndr 2022;91:73–78. 35551149 10.1097/QAI.0000000000003019 PMC9377491

[29] Rebeiro PF Emond B Rossi C. Incidence of cardiometabolic outcomes among people living with HIV-1 initiated on integrase strand transfer inhibitor versus non-integrase strand transfer inhibitor antiretroviral therapies: a retrospective analysis of insurance claims in the United States. J Int AIDS Soc 2023;26:e0. 10.1002/jia2.26123 PMC10258864 37306118

[30] Byonanebye DM Polizzotto MN Neesgaard B. Incidence of hypertension in people with HIV who are treated with integrase inhibitors versus other antiretroviral regimens in the RESPOND cohort consortium. HIV Med 2022;23:895–910. 35233903 10.1111/hiv.13273 PMC9545382

[31] Pantazis N Papastamopoulos V Antoniadou A. Changes in body mass index after the initiation of antiretroviral treatment: differences by class of core drug. Viruses 2022; 14:1677. 10.3390/v14081677 PMC9415309 36016299

[32] Nou E Lu MT Looby SE. Serum oxidized low-density lipoprotein decreases in response to statin therapy and is independently associated with reductions in coronary plaque in patients with HIV. AIDS 2016;30:583–90. 26558731 10.1097/QAD.0000000000000946 PMC5041529

[33] Gao S Zhao D Wang M. Association between circulating oxidized LDL and atherosclerotic cardiovascular disease: a meta-analysis of observational studies. Liu J Can J Cardiol. 2017;33:1624–1632. 29173602 10.1016/j.cjca.2017.07.015

[34] Zidar D.A. Juchnowski S Ferrari B. Oxidized LDL levels are increased in HIV infection and may drive monocyte activation. J Acquir Immune Defic Syndr 2015;69:154–160. 25647528 10.1097/QAI.0000000000000566 PMC4446174

[35] Parra S Coll B Aragonés G. Nonconcordance between subclinical atherosclerosis and the calculated Framingham risk score in HIV-infected patients: relationships with serum markers of oxidation and inflammation. HIV Med 2010;11:225–231. 19845792 10.1111/j.1468-1293.2009.00766.x

[36] Toribio M Fitch KV Sánchez L. Effects of pitavastatin and pravastatin on markers of immune activation and arterial inflammation in HIV. AIDS 2017; 31:797–806. 10.1097/QAD.0000000000001427 PMC5382495 28252528

[37] Grover SA Coupal L Gilmore N. Impact of dyslipidemia associated with highly active antiretroviral therapy (HAART) on cardiovascular risk and life expectancy. Mukherjee J Am J Cardiol 2005;95:586–591. 15721096 10.1016/j.amjcard.2004.11.004

[38] Hsue PY Waters DD. Time to recognize HIV infection as a major cardiovascular risk factor. hsue PY, waters DD. Circulation 2018;138:1113–1115. 30354392 10.1161/CIRCULATIONAHA.118.036211 PMC8063774

[39] Baker JV Neuhaus J Duprez D. Inflammation predicts changes in high-density lipoprotein particles and apolipoprotein A1 following initiation of antiretroviral therapy. AIDS 2011;25:2133–42. 21857489 10.1097/QAD.0b013e32834be088 PMC3320724

[40] Sviridov D Mukhamedova N Makarov AA. Comorbidities of HIV infection: role of nef-induced impairment of cholesterol metabolism and lipid raft functionality. AIDS 2020;34:1–13. 31789888 10.1097/QAD.0000000000002385 PMC6903377

[41] Carpentier A Patterson BW Uffelman KD. Mechanism of highly active anti-retroviral therapy-induced hyperlipidemia in HIV-infected individuals. Atherosclerosis 2005;178:165–72. 15585214 10.1016/j.atherosclerosis.2004.07.035

[42] Zha BS Wan X Zhang X. HIV protease inhibitors disrupt lipid metabolism by activating endoplasmic reticulum stress and inhibiting autophagy activity in adipocytes. PLoS One 2013;8:. 10.1371/journal.pone.0059514 PMC3606318 23533630

[43] Gwag T Meng Z Sui Y. Non-nucleoside reverse transcriptase inhibitor efavirenz activates PXR to induce hypercholesterolemia and hepatic steatosis. J Hepatol 2019;70:930–40. 30677459 10.1016/j.jhep.2018.12.038 PMC6462244

[44] Baker JV Sharma S Achhra AC. Changes in cardiovascular disease risk factors with immediate versus deferred antiretroviral therapy initiation among HIV-positive participants in the START (Strategic Timing of Antiretroviral Treatment) trial. J Am Heart Assoc 2017;6:0. 10.1161/JAHA.116.004987 PMC5524070 28533305

[45] Gatell JM Assoumou L Moyle G. Switching from a ritonavir-boosted protease inhibitor to a dolutegravir-based regimen for maintenance of HIV viral suppression in patients with high cardiovascular risk. AIDS 2017;31:2503–14. 29112070 10.1097/QAD.0000000000001675 PMC5690310

[46] Kamara DA Smith C Ryom L. Longitudinal analysis of the associations between antiretroviral therapy, viraemia and immunosuppression with lipid levels: the D:A:D study. Antivir Ther 2016;21:495–506. 27114439 10.3851/IMP3051

[47] Cao R Hu Y Wang Y. Prevention of HIV protease inhibitor-induced dysregulation of hepatic lipid metabolism by raltegravir via endoplasmic reticulum stress signaling pathways. J Pharmacol Exp Ther 2010;334:530–39. 20472667 10.1124/jpet.110.168484 PMC2913777

[48] Ezechi LO Musa ZA Otobo VO. Trends and risk factors for obesity among HIV positive Nigerians on antiretroviral therapy. Ceylon Med J 2016;61:56–62. 27423745 10.4038/cmj.v61i2.8300

[49] McComsey GA Moser C Currier J. Body composition changes after initiation of raltegravir or protease inhibitors: ACTG A5260s. Clin Infect Dis 2016;62:853–62. 26797215 10.1093/cid/ciw017 PMC4787610

[50] Venter WD Sokhela S Simmons B. Dolutegravir with emtricitabine and tenofovir alafenamide or tenofovir disoproxil fumarate versus efavirenz, emtricitabine, and tenofovir disoproxil fumarate for initial treatment of HIV-1 infection (ADVANCE): week 96 results from a randomised, phase 3, non-inferiority trial. Lancet HIV 2020;7:0–76. 10.1016/S2352-3018(20)30241-1 33010240

[51] Bernardino JI Mocroft A Wallet C. Body composition and adipokines changes after initial treatment with darunavir-ritonavir plus either raltegravir or tenofovir disoproxil fumarate-emtricitabine: a substudy of the NEAT001/ANRS143 randomised trial. PLoS One 2019;14:0. 10.1371/journal.pone.0209911 PMC6349314 30689664

[52] Calmy A Sanchez TT Kouanfack C. Dolutegravir-based and low-dose efavirenz-based regimen for the initial treatment of HIV-1 infection (NAMSAL): week 96 results from a two-group, multicentre, randomised, open-label, phase 3 non-inferiority trial in Cameroon. Lancet HIV 2020;7:0–87. 10.1016/S2352-3018(20)30238-1 33010241

[53] Menard A Meddeb L Tissot-dupont H. Dolutegravir and weight gain: an unexpected bothering side effect? AIDS 2017;31:1499–500. 28574967 10.1097/QAD.0000000000001495

[54] Norwood J Turner M Bofill C. Brief Report: weight gain in persons with HIV switched from efavirenz-based to integrase strand transfer inhibitor-based regimens. J Acquir Immune Defic Syndr 2017;76:527–31. 28825943 10.1097/QAI.0000000000001525 PMC5680113

[55] Bourgi K Rebeiro PF Turner M. Greater weight gain in treatment-naive persons starting dolutegravir-based antiretroviral therapy. Clin Infect Dis 2020;70:1267–74. 31100116 10.1093/cid/ciz407 PMC8205610

[56] Bourgi K Jenkins CA Rebeiro PF. Weight gain among treatment-naïve persons with HIV starting integrase inhibitors compared to non-nucleoside reverse transcriptase inhibitors or protease inhibitors in a large observational cohort in the United States and Canada. J Int AIDS Soc 2020;23:0. 10.1002/jia2.25484 PMC7159248 32294337

[57] Martínez-Sanz J Blanco JR Muriel A. Weight changes after antiretroviral therapy initiation in CoRIS (Spain): a prospective multicentre cohort study. J Int AIDS Soc 2021;24:0. 10.1002/jia2.25732 PMC8150051 34036745

[58] Galdamez R García JA Fernández M. Short-term increase in risk of overweight and concomitant systolic blood pressure elevation in treatment-naive persons starting INSTI-based antiretroviral therapy. Open Forum Infect Dis 2019;6:0. 10.1093/ofid/ofz491 PMC7047949 32128334

[59] Summers NA Lahiri CD Angert CD. Metabolic changes associated with the use of integrase strand transfer inhibitors among virally controlled women. J Acquir Immune Defic Syndr 2020;85:355–62. 33060420 10.1097/QAI.0000000000002447 PMC7577246

[60] Saums MK King CC Adams JC. Combination antiretroviral therapy and hypertensive disorders of pregnancy. Obstet Gynecol 2019;134:1205–14. 31764730 10.1097/AOG.0000000000003584 PMC7036166

[61] Domingo P Gutierrez Mdel M Gallego-Escuredo JM. Effects of switching from stavudine to raltegravir on subcutaneous adipose tissue in HIV-infected patients with HIV/HAART-associated lipodystrophy syndrome (HALS). A clinical and molecular study. PLoS One 2014;9:0. 10.1371/journal.pone.0089088 PMC3935839 24586518

[62] Martin A Moore CL Mallon PW. HIV lipodystrophy in participants randomised to lopinavir/ritonavir (LPV/r) +2-3 nucleoside/nucleotide reverse transcriptase inhibitors (N(t)RTI) or LPV/r + raltegravir as second-line antiretroviral therapy. PLoS One 2013;8:0. 10.1371/journal.pone.0077138 PMC3813715 24204757

